# Supplementary material for: Dosimetric Comparison and Selection Criteria of Intensity-Modulated Proton Therapy and Intensity-Modulated Radiation Therapy for Adaptive Re-Plan in T3-4 Nasopharynx Cancer Patients
Source: Cancers (Basel). 2024 Oct 5;16(19):3402. doi: 10.3390/cancers16193402 (PMC11476283; doi:10.3390/cancers16193402)
Supplement: Supplementary file 1 [file cancers-16-03402-s001.zip › cancers-3233921-supplementary.pdf]

Figure S1. A sample of the adaptive plans with helical tomotherapy (A) and intensity-modulated proton therapy (B).

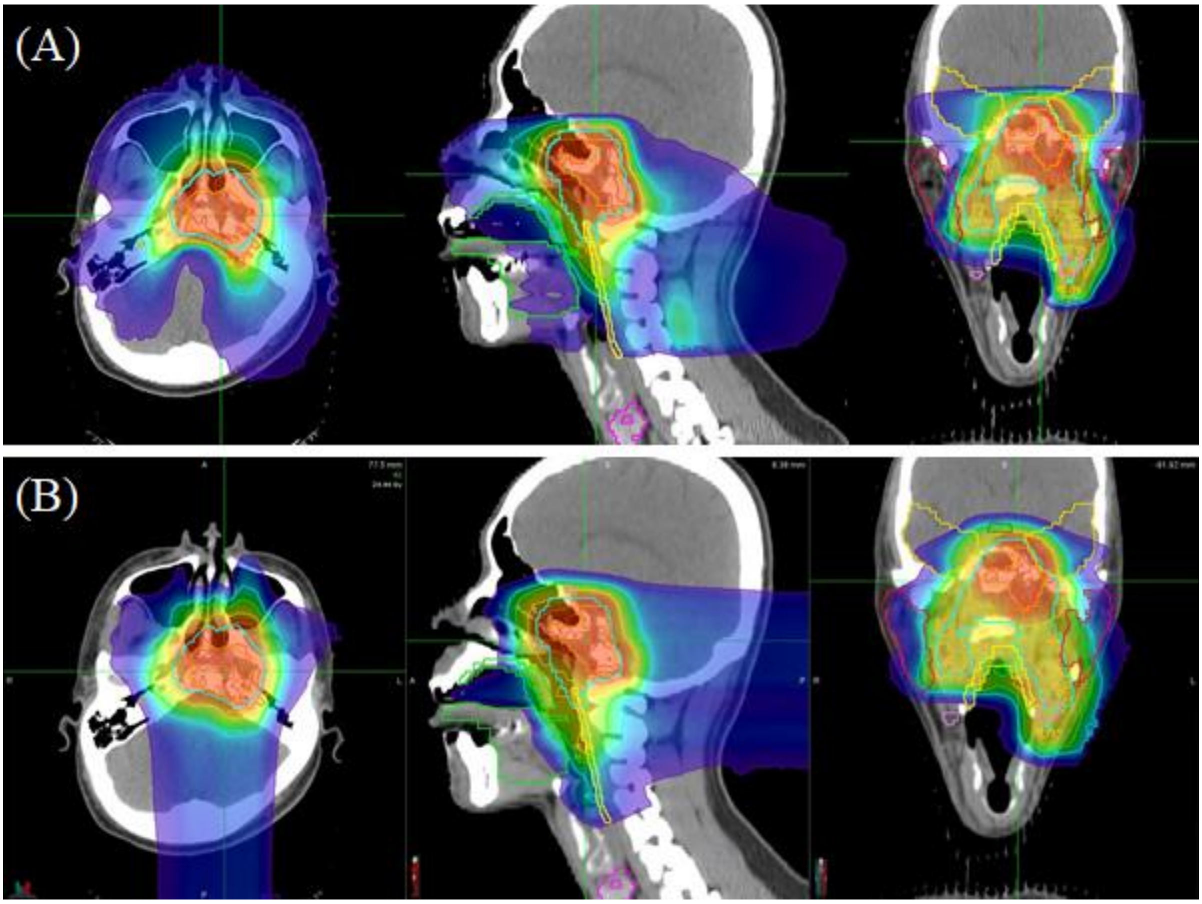

Table S1. The correlations between the target-to-OAR distance and OAR doses.

		GTV-to-OAR distance			CTV-to-OAR distance		
		Correlation coefficient	Linear regression		Correlation coefficient	Linear regression	
			Regression coefficient, B	R^2		Regression coefficient, B	R^2
Brainstem, D1	HT	-0.835	-7.301	0.698	-0.795	-10.337	0.632
	IMPT	-0.802	-6.923	0.642	-0.686	-8.811	0.470
Temporal lobes, D1	HT	-0.907	-7.333	0.823	-0.759	-14.792	0.576
	IMPT	-0.853	-6.319	0.727	-0.764	-13.651	0.584
Optic chiasm, Dmax	HT	-0.785	-8.056	0.615	-0.851	-9.903	0.724
	IMPT	-0.814	-8.971	0.663	-0.938	-11.708	0.880
Optic nerves, Dmax	HT	-0.711	-8.946	0.506	-0.921	-11.990	0.848
	IMPT	-0.737	-7.995	0.543	-0.914	-10.268	0.836
P-cord, D1	HT	-0.429	-1.780	0.184	-0.525	-3.231	0.276
	IMPT	-0.724	-5.814	0.524	-0.747	-8.890	0.558
